# Prion-like Properties of Short Isoforms of Human Chromatin Modifier PHC3

**DOI:** 10.3390/ijms26041512

**Published:** 2025-02-11

**Authors:** Daniil Kachkin, Andrew A. Zelinsky, Nina V. Romanova, Konstantin Y. Kulichikhin, Pavel A. Zykin, Julia I. Khorolskaya, Zachery J. Deckner, Andrey V. Kajava, Aleksandr A. Rubel, Yury O. Chernoff

**Affiliations:** 1 Laboratory of Amyloid Biology, St. Petersburg State University, St. Petersburg 199034, Russia; pspdaniel@mail.ru (D.K.); andrew_zelinsky@mail.ru (A.A.Z.); niro0067@student.umu.se (N.V.R.); konstantin.kulichikhin@gmail.com (K.Y.K.); 2 Department of Cytology and Histology, St. Petersburg State University, St. Petersburg 199034, Russia; pavel.zykin@spbu.ru; 3 Institute of Cytology, Russian Academy of Sciences, St. Petersburg 194064, Russia; khorolskaya@incras.ru; 4 School of Biological Sciences, Georgia Institute of Technology, Atlanta, GA 30332-2000, USA; zdeckner@oglethorpe.edu; 5 Cell Biology Research Center, UMR 5237, National Center for Scientific Research (CNRS), University of Montpellier, 34293 Montpellier, France; andrey.kajava@crbm.cnrs.fr

**Keywords:** amyloid, C-DAG, chromatin, PHC3, prion

## Abstract

The formation of self-perpetuating protein aggregates such as amyloids is associated with various diseases and provides a basis for transmissible (infectious or heritable) protein isoforms (prions). Many human proteins involved in the regulation of transcription contain potentially amyloidogenic regions. Here, it is shown that short N-terminal isoforms of the human protein PHC3, a component of the chromatin-modifying complex PRC1 (Polycomb repressive complex 1), can form prion-like aggregates in yeast assays, exhibit amyloid properties in the *E. coli*-based C-DAG assay, and produce detergent-resistant aggregates when ectopically expressed in cultured human cells. Moreover, aggregates of short isoforms can sequester the full-length PHC3 protein, causing its accumulation in the cytosol instead of the nucleus. The introduction of an aggregating short PHC3 isoform alters the transcriptional profile of cultured human cells. It is proposed that the aggregation of short isoforms is involved in the feedback downregulation of PRC1 activity, leading to more open chromatin configuration.

## 1. Introduction

A variety of proteins are capable of forming fibrous cross-β aggregates, termed amyloids [1]. In humans and other mammals, amyloids are associated with devastating diseases, such as Alzheimer’s and Parkinson’s diseases [1,2,3,4]. Infectious protein-based agents, known as prions, are typically based on amyloids and can transmit diseases such as Creutzfeldt–Jakob disease, kuru, and bovine spongiform encephalopathy (BSE), commonly referred to as “mad cow disease” [5,6]. Additionally, functional amyloids that are involved in important biological processes have been described [7,8,9]. In yeast and other fungi, amyloid-based prions control heritable traits and are transmitted via the cytoplasm [10,11,12]. Many yeast prions contain domains (termed prion domains or PrDs) that are responsible for their aggregation. These domains are frequently distinct from functional parts of the same proteins and are characterized by the presence of intrinsically disordered regions (IDRs) [10,13]. A variety of higher eukaryotic (including human) proteins, among them many transcription regulators, contain IDRs that resemble yeast PrDs [14]. Beyond their involvement in amyloid formation, these IDRs may also regulate processes such as liquid–liquid phase separation [15,16,17,18]. The functional roles of the PrD-like domains of mammalian and human proteins remain a matter of discussion.

Amyloid fibrils can be generated both in vivo and in vitro [4]. These fibrils possess distinctive characteristics that differentiate them from ordered non-amyloid protein fibrils and amorphous aggregates. These include the capacity to bind amyloid-specific dyes, such as Congo Red [19,20], and resistance to ionic detergents, including sodium lauryl sarcosinate, SLS [21]. The Congo Red staining of amyloid fibrils results in birefringence in polarized light [22,23], which can be observed in stained tissues and specialized test systems, such as C-DAG [24]. In vivo, amyloids are typically detected via their deposition in tissues at the advanced stages of diseases and/or via their functional impact in specifically designed experimental systems [9,25]. Randomized in vivo detection of amyloids by biochemical means is frequently challenging due to the small amounts of fibrils and/or the transient nature of amyloid aggregation in the cases of some functional amyloids. Algorithms for computational predictions of amyloid potential have been developed, but they still possess limited predictive power [26].

In this study, we utilized the ArchCandy algorithm [27] to identify potential amyloidogenic proteins in the human proteome. Among the identified proteins, we discovered truncated isoforms of the human Polyhomeotic-like Protein 3 (PHC3). PHC3 is a component of Polycomb repressive complex 1 (PRC1), which modulates the expression of multiple genes (including those involved in developmental processes) via chromatin modifications and packaging. It serves as one of the key regulators of transcriptional activity ontogenesis [28]. Together with its paralogs and interacting partners, PHC3 forms PcG bodies chromatin-associated protein complexes that cluster and inhibit gene expression [29,30,31,32]. A variety of species of PHC3 mRNAs, resulting from alternative splicing, can be identified in the human transcriptome [33,34]. Full-length isoforms of PHC3 and its paralogs, PHC1 and PHC2, contain the sterile alpha motif (SAM) [35], promoting protein polymerization, PRC1 clustering, and tight chromatin packaging that results in transcriptional repression [30,34]. All known information about the functions of PHC3 currently refers to its full-length isoform, while the functions of the shortened isoforms lacking the SAM remain unknown. Mutational alterations [36] to or the amplification [37] of PHC3 have been linked to various forms of cancer. PHC3 is an evolutionarily conserved protein, and its homologs are found in various multicellular animals [28,34]. Notably, PHC3 and its homologs contain IDRs with potential PrD-like composition, whose roles have not been understood previously [38].

Here, we demonstrate that short isoforms of human PHC3, which lack the functional region with the SAM domain but contain IDRs, possess prionogenic and amyloidogenic potential when expressed in yeast or bacterial cells. Notably, short isoforms of PHC3 form cytosolic detergent-resistant aggregates when ectopically expressed in human cells and promote the aggregation and mislocalization of full-length PHC3, resulting in genome-wide alterations in transcriptional regulation. These findings suggest that amyloidogenic sequences can regulate PHC3 function in chromatin packaging and transcriptional repression.

## 2. Results

### 2.1. Multiple Isoforms of PHC3 Exist in Human Cells

**Figure 1:**
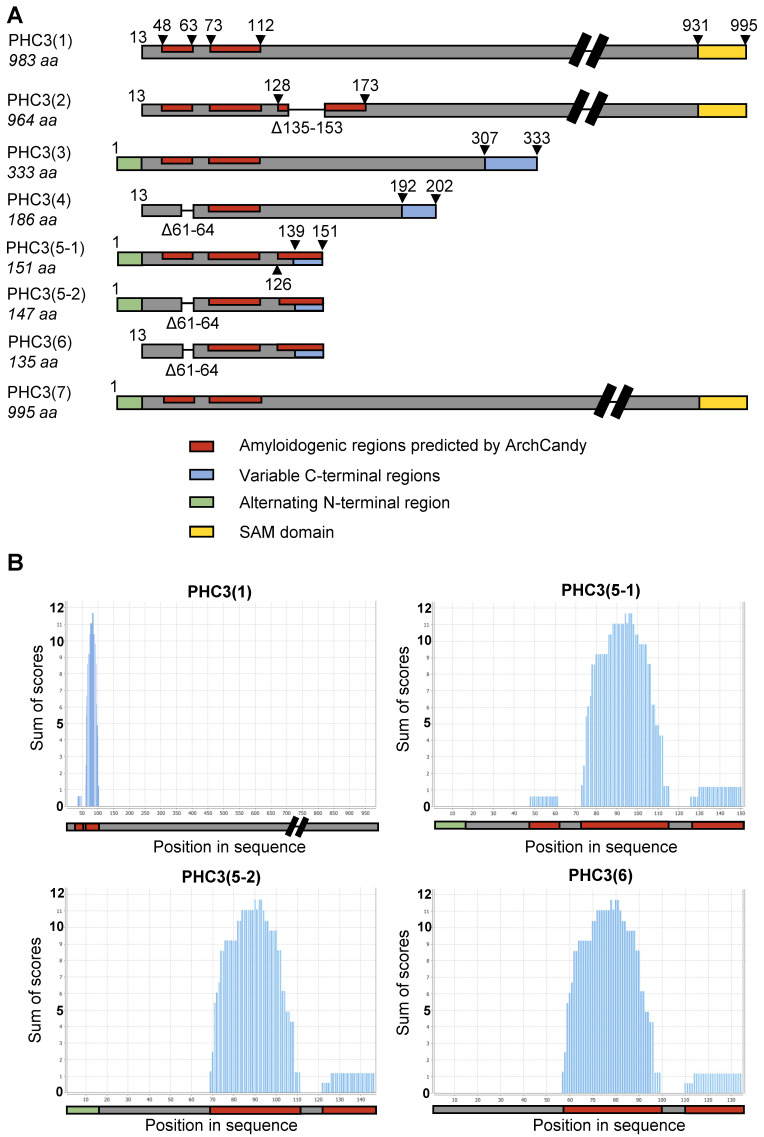
PHC3 isoforms and their predicted amyloidogenic properties. (**A**)—Known human PHC3 isoforms, with PHC3(1) being the major functional (canonical) isoform. Colors indicate the following: potentially amyloidogenic motifs predicted by ArchCandy (red); regions that are common for various PHC3 isoforms (grey); the SAM domain, involved in the homopolymerization of PHC proteins (yellow); alternating N-terminal regions (green); and variable C-terminal regions of some truncated PHC3 isoforms (blue), which differ from the reference sequence of PHC3(1). Numbers correspond to aa positions, beginning from the start of the longest isoform, PHC3(7). (**B**)—Sequences of PHC3(1), PHC3(5-1), PHC3(5-2), and PHC3(6) isoforms, analyzed by ArchCandy. The X-axis and Y-axis represent the aa positions of a PHC3 isoform, and the score reflecting the β-arch-forming propensity, respectively. A protein region is classified as potentially amyloidogenic if its score exceeds a threshold value of 0.575.

The UniProt database (uniprot.org) indicates that human cells contain mRNAs that potentially code for multiple isoforms of the PHC3 protein (UniProt ID: Q8NDX5) (Figure 1A). The full-length isoforms PHC3(1) and PHC3(7), which are 983 and 995 amino acid (aa) residues in length, respectively, differ from each other by the N-terminal stretch of 12 aa residues. These isoforms include a zinc-finger motif and a sterile alpha motif (SAM) (responsible for oligomerization) that are involved in the functioning of PHC3 as a component of a chromatin-modifying PRC complex. In contrast, the short isoforms PHC3(5), designated here and further as PHC3(5-1), and PHC3(6) encompass only the N-proximal region of PHC3 and lack functional motifs. PHC3(6), comprising 135 amino acid residues, differs from PHC3(5-1) (151 aa length) by the absence of the N-terminal 12 aa stretch and the internal 4 aa region (Δ61–64). The PHC3(5-1) and PHC3(6) isoforms were successfully isolated from the cDNA library made from HEK293T cells. Additionally, a novel isoform, designated PHC3(5-2), was identified. It includes the N-terminal 12 aa stretch but lacks the internal 4 aa region (Δ61–64), thus combining some features of isoforms 5-1 and 6 (Figure 1A).

### 2.2. PHC3 Isoforms Contain Potentially Amyloidogenic Regions

**Figure 2:**
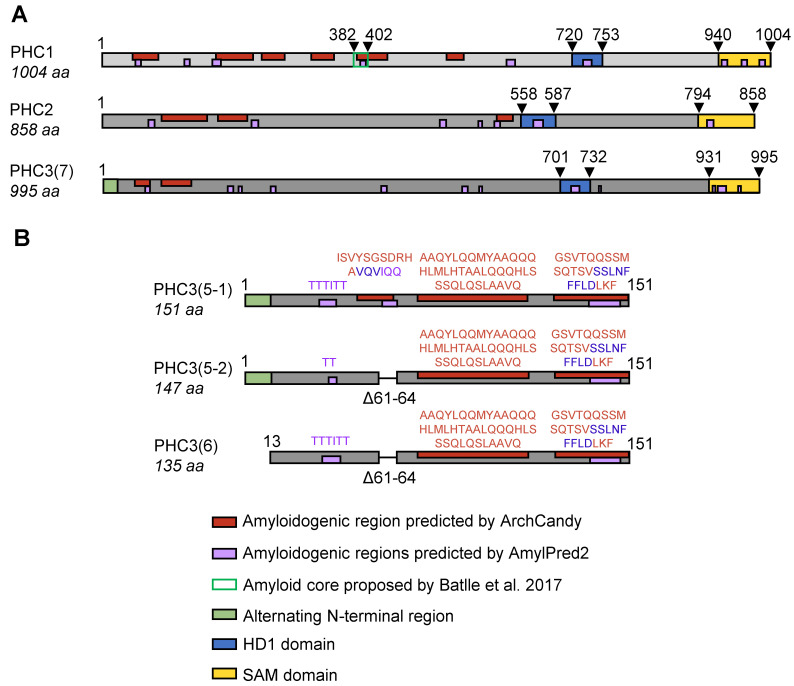
Comparison of predicted amyloidogenic regions among human PHC proteins and isoforms. (**A**)—Comparison of PHC1, PHC2, and PHC3 proteins with domains and predicted amyloidogenic motifs indicated. (**B**)—Comparison of ArchCandy and AmylPred2 predictions for short PHC3 isoforms. Sequences on top of respective regions correspond to motifs predicted by ArchCandy (in red), AmylPred2 (in magenta), or both (in blue). Numbers correspond to aa positions. Regions predicted by ArchCandy and AmylPred2, as well as N-terminal regions, HD1, and SAM domains are shown in red, magenta, green, blue, and yellow boxes, respectively. The amyloid core proposed in Ref. [38] is shown within a square with a green outline.

At the subsequent stage of analysis, the ArchCandy algorithm [27] was utilized to predict the superpleated “β-arch” structures (characteristic of numerous amyloids) in the context of the PHC3 sequence. The analysis identified three motifs that could be amyloidogenic, at positions 48–61, 73–112, and 126–151, located within the isoform 5-1 (Figure 1A,B). The first two motifs are also present in the full-length isoform PHC3(1), while isoforms PHC3(5-2) and PHC3(6) contain the last two motifs but lack the amyloidogenic region 48–61, which is disrupted by an internal deletion (Figure 1B). Notably, PHC2 [33], a distant paralog of PHC3, exhibits homology to PHC3, including the region that overlaps with amyloidogenic motifs predicted by ArchCandy (Figure 2A and Table S1). An analysis of the PHC2 sequence by ArchCandy confirms the amyloidogenic potential of this region and identifies other potentially amyloidogenic motifs elsewhere in the protein (Figure 2A). Another paralog of PHC3, PHC1 [34], is more divergent from PHC2 and PHC3 within its N-proximal region; however, it still contains several amyloidogenic motifs identified by ArchCandy (Figure 2A). One of these motifs overlaps with the region showing an alignment between all three proteins (Table S1), while another motif (Figure 2A) overlaps with the amyloidogenic peptide, which was detected in PHC1 by Batlle et al. [38] using another computational approach, pWALTZ [39]. The aggregation of this peptide (separated from the rest of the protein) was also confirmed in vitro [38]. An analysis of the protein sequences using another amyloid prediction algorithm, AmylPred2 [40], also identified multiple amyloidogenic stretches within PHC1, PHC2, and PHC3 (Figure 2A). Some of the AmylPred2-predicted stretches, which are localized within the region encompassed by the short PHC3 isoforms (5-1, 5-2, and 6), overlap with amyloidogenic motifs predicted by ArchCandy (Figure 2B). Overall, our data show that PHC proteins contain multiple potentially amyloidogenic motifs and that the most pronounced amyloidogenic motifs of PHC3 are present in its short isoforms, which lack functional domains.

### 2.3. PHC3 Isoforms Possess Prion-Nucleating Properties in Yeast Cells

**Figure 3:**
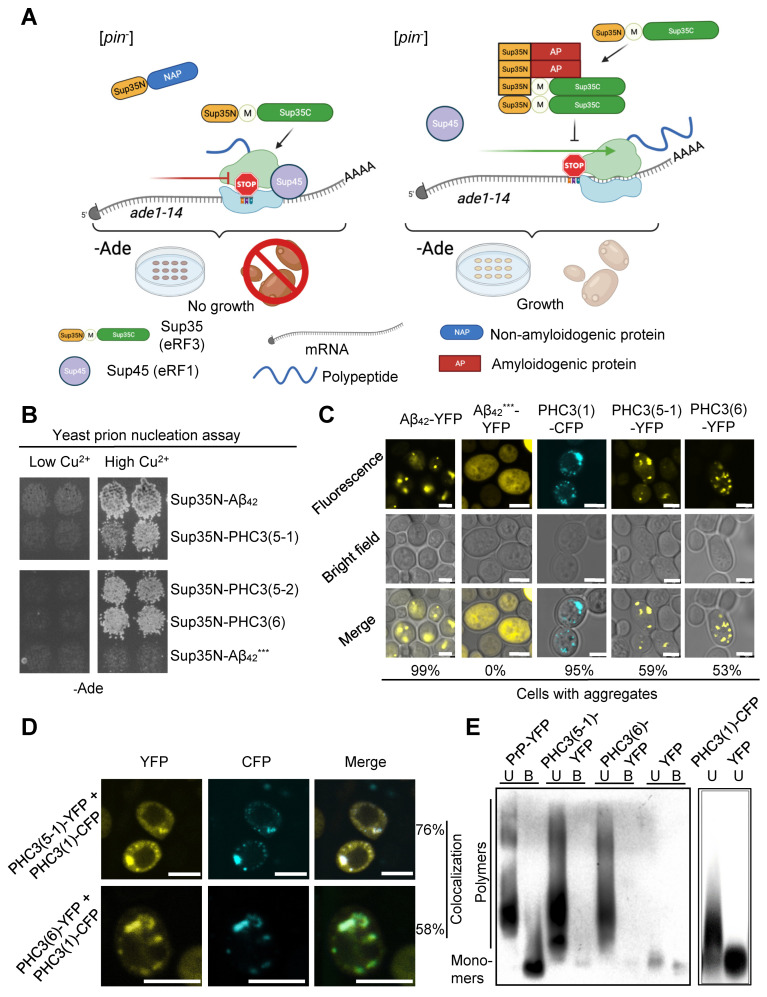
Prion-like properties of short PHC3 isoforms in yeast assays. (**A**)—Yeast phenotypic assay for de novo prion nucleation. The protein sequences tested are fused to the prion (N) domain of the Sup35 (eRF3) translation termination (release) factor. Typically, Sup35N does not nucleate the prion state in the absence of other prions. If the fused protein sequence is amyloidogenic (AP), it promotes prion nucleation. The full-length Sup35 protein is then immobilized into aggregates and partially inactivated, leading to the readthrough of the *ade1-14* (UGA) reporter mutation. As a result, the cells become able to grow on a medium that lacks adenine. Sup35N, M, and C refer to the Sup35N-terminal (prion), middle, and C-proximal (functional) domains, respectively; [*pin*^−^] strain is a yeast strain lacking other pre-existing prions; −Ade—selective synthetic medium lacking adenine. (**B**)—Isoforms 5-1, 5-2, and 6 of the human PHC3 protein, fused to Sup35N, are capable of nucleating the Sup35 prion ([*PSI^+^*]) in the yeast phenotypic assay, described in panel A. Two independent representative transformants are shown for each plasmid (typically, no less than 8 transformants are tested). Chimeric constructs are expressed from the copper-inducible (*P_CUP1_*) promoter in the presence of 100 µM CuSO_4_, followed by velveteen replica plating onto the −Ade medium. Sup35N-Aβ_42_ is used as a positive control, while Sup35N-Aβ_42_***, based on the Aβ_42_ derivative with triple mutation F19S/F20S/I31P that blocks Aβ_42_ aggregation and prion nucleation [41], is used as a negative control. −Ade plates are incubated for 7 days. The upper and bottom portions of the panel are carved from the image of one and the same plate. (**C**)—Fluorescence microscopy of PHC3(1)-CFP, PHC3(5-1)-YFP, and PHC3(6)-YFP constructs, expressed in yeast cells from the *P_CUP1_* promoter in the presence of 100 µM CuSO_4_. Chimeric proteins Aβ_42_-YFP and Aβ_42_***-YFP are used as positive and negative controls, respectively. The percentage of cells with puncta (aggregates) out of all cells with fluorescence is shown under the images. Scale bar is 3 μm. See Table S2 for numbers. (**D**)—Coaggregation of short isoforms PHC3(5-1) and PHC3(6), fused to YFP, with full-length protein PHC3(1), fused to CFP. The conditions of the experiment are the same as those in panel **C**. The percentage of cells showing colocalization out of all cells containing both types of puncta is shown. Scale bar is 5 µm. See Table S3 for numbers. (**E**)—Detergent-resistant aggregates of short PHC3 isoforms, fused to YFP, and of full-length PHC3 protein, fused to CFP, are detected by semi-denaturing detergent agarose gel electrophoresis (SDD-AGE), followed by Western blotting and reaction to GFP-specific antibodies (also recognizing YFP and CFP). Human prion protein PrP fused to YFP and YFP itself, expressed in yeast cells, are used as positive and negative controls, respectively. Sodium lauryl sarcosinate (SLS) is added to each sample at the final concentration of 3%. U and B refer to unboiled and preboiled samples, respectively (preboiling solubilizes detergent-resistant aggregates).

Next, we checked if the amyloidogenic potential of PHC3 isoforms, predicted by computational analysis, could be detected in an experiment. First, we used a yeast phenotypic assay, described in our previous work [41], that employs the fusion of a potential amyloidogenic protein or peptide to the prion domain (Sup35N) of the yeast translation termination factor Sup35 (Figure 3A). Sup35N, even when expressed at high levels, cannot efficiently initiate the formation of the Sup35 prion in [*pin*] yeast cells, lacking other pre-existing prions. However, the polymerization of a fused amyloidogenic protein promotes the polymerization of Sup35N, which subsequently converts into a prion state and recruits full-length Sup35 into prion polymers. As a result, the process of translation termination is impaired, which can be detected by growth on the medium lacking adenine (-Ade) and a light-pink (as opposed to red) color on the complete medium of the yeast strain containing a UGA nonsense mutation within the *ADE1* gene (*ade1-14*) (Figure 3A). Indeed, the attachment of any short PHC3 isoform (5-1, 5-2, or 6) to Sup35N promoted the formation of the Sup35 prion ([*PSI^+^*]), conferring growth on –Ade. This result was comparable to the one observed with the chimeric construct, composed of Sup35N fused to human amyloidogenic protein Aβ_42_, which served as a positive control (Figure 3B). As isoform 5-2 combines certain properties of the isoforms 5-1 and 6, our subsequent analysis was focused on the latter two isoforms.

To eliminate the potential impact of the Sup35N sequence, we fused short PHC3 isoforms, as well as the full-length isoform, to either yellow (YFP) or cyan (CFP) fluorescent protein, and studied their aggregation in yeast cells by fluorescence microscopy. The majority of cells exhibited puncta formation, which indicates possible aggregation (Figure 3C and Table S2). Moreover, when the full-length isoform PHC3(1), tagged with CFP, was coexpressed with either PHC3(5-1) or PHC3(6), tagged with YPF, the majority of the cyan and yellow puncta colocalized (Figure 3D and Table S3), suggesting that long and short isoforms of PHC3 interact with each other. Finally, semi-denaturing detergent agarose gel electrophoresis (SDD-AGE) revealed that aggregates, formed by short, YFP-tagged PHC3(5-1) or PHC3(6) isoforms, or by the full-length, CFP-tagged PHC3(1) isoform, in yeast cells are composed of detergent-resistant polymers (Figure 3E), as is typical of amyloid-based endogenous yeast prions. Altogether, these data show that short isoforms of PHC3 exhibit prion-like properties in yeast assays, while a full-length isoform is capable of aggregating and colocalizing with the aggregates of short isoforms.

### 2.4. Short Isoforms of PHC3 Form Amyloid Fibrils in the C-DAG Assay

**Figure 4:**
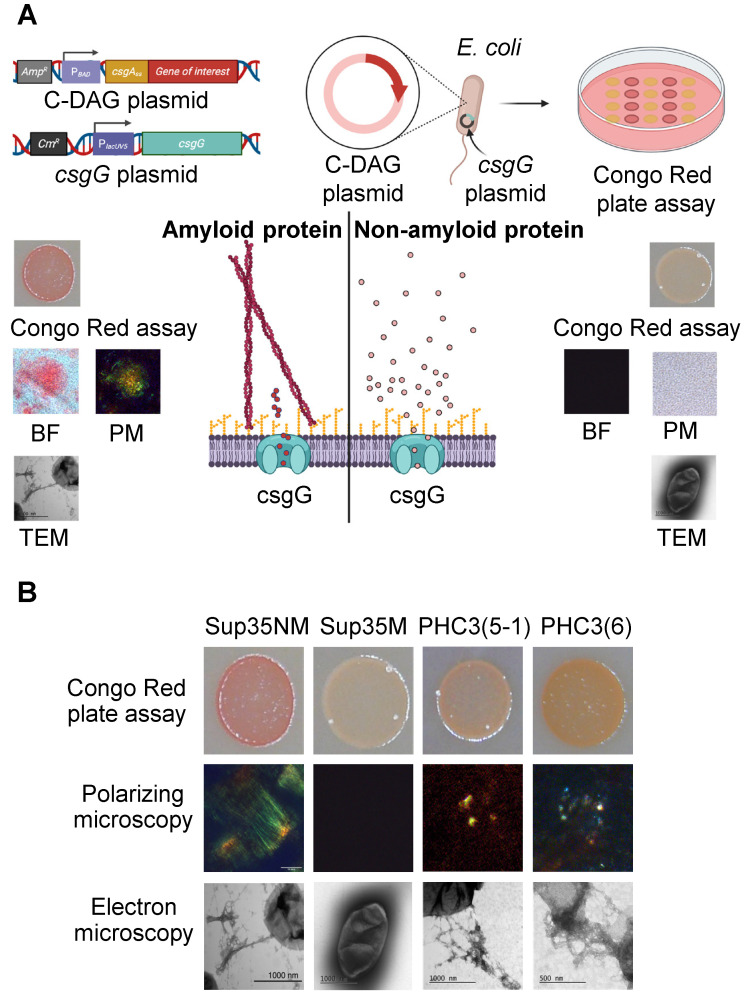
Amyloid formation by short PHC3 isoforms in *E. coli*-based C-DAG assay. (**A**) Scheme of the C-DAG assay. The protein of interest is fused to the signal sequence of the *E. coli* csgA protein and excreted through the csgG pore. If this protein forms amyloid fibrils on the surface of bacterial cells, bacterial cells can be stained by the amyloid-binding dye Congo Red (added to the growth medium), as detected on plates and in a bright field (BF), and show birefringence in polarized light (PM). Fibrils attached to bacterial cells through csgG pores can also be visualized by transmission electron microscopy (TEM). (**B**) Results of the C-DAG assay for the short PHC3 isoforms 5-1 and 6, which exhibit Congo Red staining and birefringence and show the formation of amyloid fibrils, detectable by TEM. The amyloid-forming fragment of Sup35 (Sup35NM) and its middle region alone, not capable of forming an amyloid (Sup35M), are used as positive and negative controls, respectively.

We utilized the Curli-dependent amyloid generator, C-DAG assay [24] to determine whether short isoforms of PHC3 can form amyloids when expressed in *E. coli*. This assay is based on the use of a chimeric construct, in which a protein of interest is fused to the N-terminal part of the bacterial curli protein csgA (Figure 4A) and exported through the outer membrane pore formed by the csgG protein. If a protein of interest forms amyloid fibrils, it remains attached to the surface of a bacterial cell. A variety of approaches can be employed to readily detect these fibrils. We have demonstrated that bacterial cells expressing the csgA-PHC3(5-1) and csgA-PHC3(6) fusion proteins are stained by the amyloid-binding dye Congo Red, exhibiting birefringence under polarized light similar to the control csgA-Sup35NM protein. This birefringence is characteristic of amyloids (Figure 4B). The observation is that birefringence in polarized light encompasses not only yellow–green, but also other shades (for example, blue and red). This observation aligns with data for other amyloids [22] and with the conclusions of the International Society of Amyloidosis (ISA) nomenclature committee, as noted in the proceedings of the 17th International Symposium on Amyloidosis [4]. Additionally, transmission electron microscopy (TEM) was employed to detect the presence of PHC(5-1) and PHC3(6) fibrils (Figure 4B).

### 2.5. Short Isoforms of PHC3 Aggregate in Human Cells and Affect the Intracellular Localization of Full-Length PHC3

**Figure 5:**
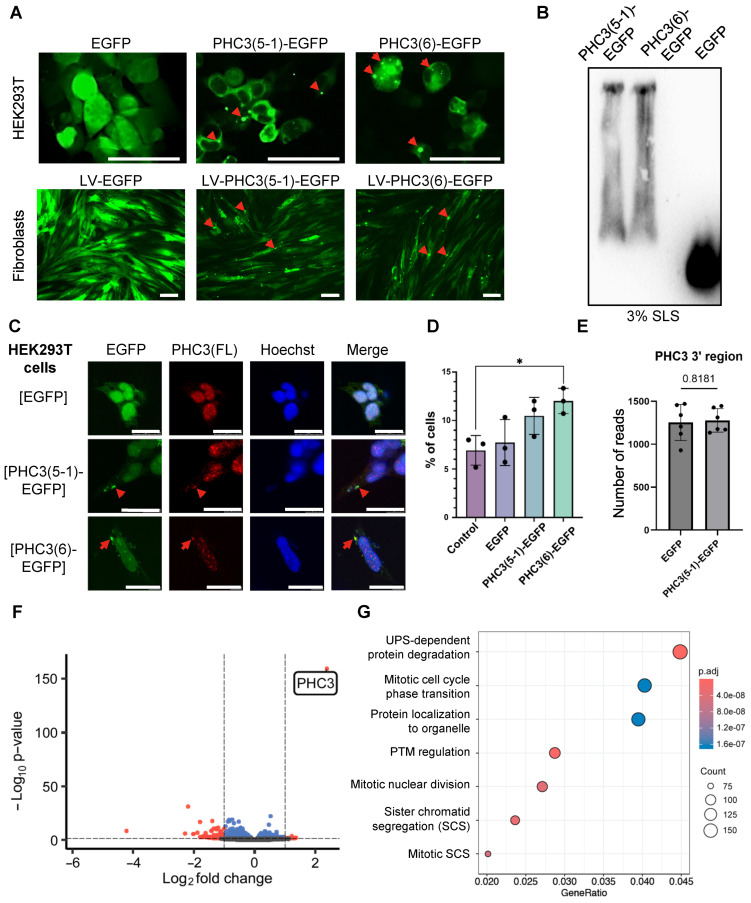
Aggregation of short PHC3 isoforms in cultured human cells. (**A**)—Fluorescence microscopy of cultured human cells, expressing PHC3(5-1) or PHC3(6) isoforms fused to EGFP, as compared to EGFP control. HEK293T cells were transfected with respective plasmids, while human fibroblasts were transduced with respective lentiviral (LV) vectors. Red arrows point to examples of puncta, corresponding to protein aggregates. Scale bar is 100 μm. (B)—Detection of detergent-resistant aggregates of short PHC3 isoforms (fused to EGFP) in HEK293T cells by SDD-AGE, followed by Western blotting and reaction to GFP-specific antibodies. HEK293T cells expressing EGFP were used as a control; 3% SLS was added to the samples. (C)—The effect of the overproduction of short PHC3 isoforms in HEK293T cells on the localization of the endogenous full-length human PHC3(1) protein. Nuclei were stained by Hoechst (blue) and full-length PHC3(1) by Alexa647 (red); PHC3(5-1)-EGFP is green. EGFP was used as a control. Red arrows point to examples of cytosolic puncta of PHC3(5-1)-EGFP or PHC3(6)-EGFP, colocalized with PHS3(FL). Scale bar is 25 μm. (D)—Proportion of HEK293T cells with cytoplasmic localization of full-length PHC3(1) in cultured HEK293T cells producing EGFP, PHC3(5-1)-EGFP, or PHC3(6)-EGFP, as well as in non-transfected (control) cells. Each dot represents the mean value of one biological replica; graphs show the overall mean and standard deviations. Statistically significant differences according to the Mann–Whitney U test (*p* < 0.05) are shown by an asterisk. For numbers, see Table S4. (E,F)—Results of sequencing analysis of the transcriptomes of HEK293T cells with PHC3(5-1)-EGFP overexpression as compared to samples overexpressing EGFP alone. Six biological repeats were analyzed in each set. The number of sequencing reads in the 3′ region of the PHC3 gene (chr 3: 170171330-170181733 bp) showed no statistically significant difference between these samples, with *p* = 0.8181 according to the *t*-test (**E**), while a Volcano plot of differential gene expression analysis shows PHC3 as one of the genes with the highest increase in expression (**F**), confirming the overexpression of 5’ proximal PHC3 isoforms. In panel F, red and blue dots represent genes with at least two-fold difference in expression, and genes with statistically significant differences between samples but less than two-fold, respectively. For details, see Datasets S1 and S2. (**G**)—Gene ontology (GO) analysis of differentially expressed genes. The X-axis visualizes a gene ratio, the color represents the adjusted *p*-value of GO terms, and the size of the circle demonstrates the number of genes. UPS—ubiquitin–proteasome system; PTM—post-translational protein modification; SCS—sister chromatid segregation. Detailed information can be found in Dataset S3.

As short isoforms of PHC3 are capable of forming amyloid-like aggregates when expressed in yeast or on the surface of a bacterial cell, we investigated whether these isoforms could aggregate in human cells. To achieve this, we fused the region coding for either PHC3(5-1) or PHC3(6) to the sequence coding for enhanced green fluorescent protein (EGFP) and placed it under the control of the cytomegalovirus promoter (*P_CMV_*). These constructs were then introduced into human cells. HEK293T cells were transfected with the pLenti-CMV-PHC3(5-1)-EGFP and pLenti-CMV-PHC3(6)-EGFP plasmids, while human fibroblasts (hFBs) were transduced with lentiviral vectors LV-CMV-PHC3(5-1)-EGFP and LV-CMV-PHC3(6)-EGFP. The aggregation of the EGFP-tagged PHC3 isoforms was detected by fluorescent microscopy (Figure 5A) in all cell lines that were transfected or transduced. The analysis of HEK293T cell extracts by SDD-AGE shows that aggregates of both PHC3(5-1)-EGFP and PHC3(6)-EGFP are composed of detergent-resistant polymers (Figure 5B) that are typical of amyloids and similar to aggregates produced by these proteins in yeast cells.

The full-length endogenous PHC3 protein is primarily located within the nuclei of human cells, as confirmed by our experiments employing secondary immunofluorescence (Figure 5C). In contrast, the EGFP-tagged short isoforms PHC3(5-1) and PHC3(6) are primarily cytoplasmic (Figure 5C). To investigate whether the presence of short isoforms affects the localization of full-length PHC3, HEK293T cells expressing PHC3(5-1)-EGFP or PHC3(6)-EGFP (along with control cells expressing EGFP) were immunostained with antibodies specific to the full-length PHC3 isoform. We observed an increased proportion of cells exhibiting the cytoplasmic localization of full-length PHC3 in cultures expressing short isoforms (Figure 5C,D). In some cases, the colocalization of aggregated short isoforms with cytoplasmic full-length PHC3 was evident (Figure 5C). In the case of PHC3(6)-EGFP overexpression, the increase in the proportion of cells with the cytosolic localization of full-length PHC3 was statistically significant, as compared to the control (Figure 5D). These findings suggest that aggregates formed by short PHC3 isoforms may sequester full-length PHC3, thereby trapping it in the cytoplasm.

### 2.6. Overproduction of PHC3(5-1)-EGFP Alters Transcription in Human Cells

The PHC3 protein is an important component of the PRC1 complex responsible for chromatin compaction and the regulation of gene expression. Truncated PHC3 isoforms lack the functional SAM domain involved in interactions with other PRC1 complexes but possess amyloid-like aggregation potential with a tendency to sequester functional PHC3 proteins in the cytoplasm. To understand whether the overproduction and aggregation of the truncated form of PHC3(5-1) can affect gene expression in human cells, we examined the transcriptome of cells overproducing PHC3(5-1)-EGFP and compared them with cells overproducing EGFP alone (see Dataset S1). Notably, the increased expression of plasmid-based short isoforms of PHC3 (but not of chromosomally encoded full-length PHC3) was confirmed by our analysis (Figure 5E,F). We identified 2427 genes with altered expression, including 53 genes with more than a two-fold change in expression level (Figure 5F and Dataset S2). Expression changes were in both directions; however, for most genes with more than a two-fold change in expression, mRNA levels were decreased in cells overproducing PHC3(5-1)-EGFP, compared to in EGFP overproducers (Figure 5F).

To understand which processes might be affected in PHC3(5-1)-EGFP-overproducing cells, we performed a gene ontology enrichment analysis of the differentially expressed genes. Notably, we noticed alterations in expression levels for a variety of genes controlling the ubiquitination and proteasome-mediated proteolysis pathway (Figure 5G and Dataset S3). Among the 86 genes with statistically significant alterations in expression that belong to this group, mRNA levels were increased for 44 genes and decreased for the rest of them in response to PHC3(5-1) overproduction. Other groups of genes with altered expression included those related to cell cycle regulation, sister chromatid segregation, and nuclear division (Figure 5G).

## 3. Discussion

Several lines of evidence suggest that short N-proximal isoforms of PHC3 (5-1, 6, and the less extensively studied isoform 5-2) exhibit amyloidogenic and prionogenic properties. The ArchCandy algorithm and other amyloid prediction tools indicate the presence of amyloidogenic sequences within IDRs, which are included in these isoforms (Figure 1B and Figure 2). Short isoforms promote prion induction in a yeast-based assay when fused to the PrD of the yeast protein Sup35 (Figure 3B), form detergent-resistant aggregates in yeast cells when fused to fluorophores (Figure 3C,D), and form fibrils with amyloid properties (as evidenced by Congo Red binding and birefringence) in the *E. coli*-based C-DAG assay (Figure 4B). Additionally, fluorophore-tagged short isoforms produce cytosolic detergent-resistant aggregates when ectopically expressed in cultured human cells (Figure 5A,B). The full-length PHC3 protein can sometimes be trapped in cytosolic aggregates of short PHC3 isoforms, as observed both in yeast cells (Figure 3C) and occasionally in human cells (Figure 5C,D). Notably, the expression and aggregation of short isoforms in human cells lead to massive alterations in the transcriptome, resulting in either an increase or decrease in the levels of multiple mRNAs (Figure 5F,G, and Datasets S1–S3). The genes affected are involved in a variety of important cellular processes, including the ubiquitin-mediated proteolysis and control of cell division (Figure 5G). This effect on gene expression is consistent with the partial depletion of full-length functional PHC3 from the nuclei of some cells accumulating cytosolic aggregates of short isoforms. Somewhat surprisingly, for genes displaying the highest level of alteration, transcription is typically reduced rather than enhanced in cells producing elevated levels of the truncated PHC3 isoform (Figure 5F). This appears counterintuitive, as the depletion of full-length PHC3 (and potentially other PHCs, as discussed below) in nuclear PRC1 complexes, which could result from their trapping in cytosolic aggregates, would be anticipated to cause a decline in PRC1 clustering and chromatin unpacking and, respectively, the derepression of transcription. However, it is possible that chromatin unpacking increases the expression of some genes coding for repressor proteins, which may in turn repress other genes. Thus, chromatin unpacking may lead to a compensatory response that overcomes the impact of reduced PRC1 clustering and decreases transcription for some sets of genes. Some of the observed changes, for example, alterations in the expression of proteolytic machinery genes (Figure 5G and Dataset S3), may represent a functional response to aggregate accumulation rather than a direct effect of chromatin unpacking. Indeed, about half of the ubiquitin–proteasome pathway genes exhibit increased mRNAs levels in the presence of overexpressed short PHC3 isoforms. It should also be noted that our approach could overlook some changes in transcription, as we used nonhomogeneous cell cultures for the transcriptome analysis. Indeed, only a fraction of cells (usually 60–80%) in our sample contain the PHC3(5-1)-EGFP plasmid. Therefore, some changes in expression could have been masked or diminished by the presence of transcripts at normal levels in cells lacking the plasmid.

Overall, our data point to the possibility of short isoforms of PHC3 being capable of regulating the function of the full-length protein via cytosolic aggregation, possibly promoting protein mislocalization and affecting the transcription of multiple genes. Notably, other PHC proteins (PHC1 and PHC2) contain regions corresponding to the short amyloidogenic isoforms of PHC3, suggesting that these proteins could potentially also be sequestered into cytosolic aggregates. It is possible that the “unclustering” of PRC1, due to the sequestration of PHC3 (and possibly other PHCs) by short isoforms, serves as a feedback regulatory mechanism, modulating the levels of PHC3 itself and overall chromatin packaging in the cell. An attractive hypothesis is that the production (and perhaps aggregation) of short PHC3 isoforms could be modulated by physiological conditions, making them potential tools for the physiological regulation of chromatin packaging. Further studies are needed to address these scenarios.

Previous data indicated that a decreased level of PHC3 serves as an oncomarker [42], while the mutant allele coding for this protein is associated with a decreased viability of patients with osteosarcoma [36]. On the other hand, the amplification of the *PHC3* gene is associated with epithelial cancer [37]. This generates an apparent contradiction, as both the hypofunction and hyperfunction of PHC3 show an association with cancer. However, it has been shown previously that the overproduction of yeast prion-forming proteins promotes their aggregation and prion formation, resulting in an ultimate decrease in their function [10]. It is, therefore, possible that *PHC3* amplification increases the level of short isoforms, leading to the more efficient aggregation and/or sequestration of the full-length PHC3 protein and thus decreasing its function in chromatin clustering.

A high abundance of PRD-like IDRs among transcriptional factors and chromatin remodelers suggests that aggregation-based regulatory networks could be widespread [43]. The formation of heritable prions by the yeast chromatin remodelers Swi1 [44] or Snt1 [45] has been described in the past. Plant cells also contain transcriptional regulators modulated by the formation of aggregates or biomolecular condensates [13,46]. Our data point to the possibility of such mechanisms also applying to human proteins. As PHC proteins, including their potentially amyloidogenic regions, are conserved among multicellular animals [28,34], it is possible that such an aggregation-based regulatory mechanism is conserved in evolution as well. Thus, our data are consistent with the potential existence of a two-level epigenetic regulation circuit in animal cells based on the self-perpetuating protein aggregation that modulates chromatin packaging and gene transcription.

## 4. Materials and Methods

### 4.1. Bacterial and Yeast Strains, and Cultivation Conditions

For plasmid construction and the amplification of plasmid DNA, *Escherichia coli* strain XL10-Gold (Stratagene, La Jolla, CA, United States) of the following genotype—*tet^R^Δ* (*mcrA*)183 Δ(*mcrCB-hsdSMR-mrr*)173 *endA1 supE44 lac96 relA1 Hte* [*F’proAB lacIqZDM15 Tn10 (tet^R^) Amy Cam^R^*]—was used for plasmid construction isolation. *E. coli* strain VS39 [24] of the following genotype—[*araD1*39] B/r Δ*(argF-lac)*169 *λ-e14-flhD5301 Δ(fruK-yeiR)*725 *(fruA25) relA1 rpsL150* (*strR*) *rbsR22 Δ(fimB-fimE)*632*(::IS1) deoC1 ΔcsgA ΔcsgB ΔcsgC ΔcsgG* [*PlacUV5-csgG; Cm*R]—was used for the C-DAG assay. Standard techniques were used for *E. coli* cultivation, transformation, and plasmid DNA extraction [47]. *E. coli* cultures were typically incubated at 37 °C unless stated otherwise.

*Saccharomyces cerevisiae* strain GT409 of the genotype *MAT*a *ade1-14 his3-Δ200 leu2-3,112 lys2 trp1-Δ ura3-52* [*psi^−^*] [*pin^−^*] [48] was used for the prion nucleation assay, while strain BY4742 of the genotype *MAT*α *his3Δ-1 leu2Δ lys2Δ ura3Δ* [*psi^−^*] [*PIN^+^*] [49] was used for fluorescence microscopy and SDD-AGE. Yeast strains were grown on YEPD organic media, or synthetic complete (SC) and selective media based on YNB (Sigma-Aldrich, St. Louis, MO, USA) [50]. Yeast cultures were typically incubated at 30 °C, unless stated otherwise. Yeast transformation was carried out according to the standard protocol [51]. To increase the expression level of constructs placed under the copper-inducible *P_CUP1_* promoter, CuSO_4_ (PanReac, USA) was added to the media to a final concentration of 150 μM.

### 4.2. Human Cell Lines and Cultivation Conditions

In this study, we utilized HEK293T cells and cultured human fibroblasts (hFBs) at passages 4 through 7. The HEK293T cells were sourced from the Russian Collection of Cell Cultures at the Institute of Cytology, Russian Academy of Sciences. The hFB cells were generously provided by K.E. Zhurenkov from the Cell Technologies Center of the Institute of Cytology, Russian Academy of Sciences. The HEK293T cell culture was maintained in a DMEM medium, while hFBs were maintained in DMEM/F12. Both media were supplemented with 10% FBS, 100 μg/mL gentamicin (Gibco, Waltham, MA, USA), and 1× GlutaMax (Gibco, Waltham, MA, USA) in a humidified incubator at 5% CO_2_ and 37 °C. All cells were passaged by adding 0.5 to 1 mL of the TripLE Express Enzyme Reagent (Gibco, USA) to cells that had been washed free of media, followed by incubation at 37 °C for 5 to 10 min. Once the cells began to detach, the TripLE reagent was removed, and the appropriate medium was added.

### 4.3. Algorithms for Prediction of Amyloidogenic Sequence

The bioinformatic algorithms ArchCandy [27] and AmylPred2 (http://aias.biol.uoa.gr/AMYLPRED2/; accessed on 26 December 2024) [40,52] were used to analyze the amyloidogenic potential of the studied protein sequences. In the settings of the ArchCandy program, during the analysis, the Scoring Threshold was set to 0.575, as recommended [27].

### 4.4. Plasmid and Viral Vectors

The plasmid and viral vectors used in this work are listed in Table S5 and described below.

#### 4.4.1. Plasmids Used to Analyze Aggregation Properties of PHC3 Isoforms in Yeast

Yeast low-copy (centromeric) plasmids pCUP1-Sup35N-Aβ_42_ and pCUP1-Sup35N-Aβ_42_***, containing, respectively, the wild-type coding sequence of the human Aβ_42_ peptide (associated with Alzheimer’s disease) and its triple-mutant derivative (F19S/F20S/I31P) incapable of amyloid formation, each fused in frame to the C-terminus of the Sup35 prion domain (Sup35N) and placed under the control of copper-inducible P_CUP1_ promoter, were described earlier [41]. The coding sequences for the PHC3(5-1), PHC3(5-2), and PHC3(6) isoforms were PCR-amplified from the cDNA library generated from human HEK293T cells and kindly provided by V.R. Ginanova (St. Petersburg State University, Russia), using specific primers PHC3(5)-EcoRI-For, PHC3(5,6)-NotI-Rev, and PHC3(6)-EcoRI-For (see Table S6), which included recognition sites for the restriction endonucleases EcoRI and NotI. The amplified fragments were subsequently digested with EcoRI and NotI and inserted into the EcoRI- and NotI-digested pCUP1-Sup35N-Aβ_42_ plasmid, replacing the Aβ_42_ encoding sequence with PHC3 isoform sequences. The resulting plasmids were designated as pCUP1-Sup35N-PHC3(5-1), pCUP1-Sup35N-PHC3(5-2), and pCUP1-Sup35N-PHC3(6).

The multicopy plasmids pCUP1-YFP-PHC3(5-1) and pCUP1-YFP-PHC3(6), bearing the sequences that encode human PHC3 isoforms, were fused in frame with a yellow fluorescent protein (YFP) under the control of the *P_CUP1_* promoter. The plasmids were constructed by replacing the *Sfi*I fragment of the pCUP1-YFP(-2)-INTctd plasmid with a PCR fragment that encodes PHC3(5-1) or PHC3(6) flanked with asymmetrical restriction site *Sfi*I. The PHC3 isoforms were amplified from the plasmids pCUP1-Sup3N-PHC3(5-1) and pCUP1-Sup3N-PHC3(6) using the primers PHC3(5)-SfiI-For and PHC3(5)-SfiI-Rev (see Table S6).

The centromeric pCUP1-PHC3(1)-CFP plasmid, derived from pRS316 [53], encodes the full-length PHC3(1) isoform fused in frame with a cyan fluorescent protein (CFP) under the control of the *P_CUP1_* promoter. The full-length PHC3 isoform was amplified from a cDNA library, kindly provided by A.Y. Aksenova, St. Petersburg State University, Russia, and produced from the human neuroblastoma cell line IMR-32 (ATCC, USA). For the amplification of the PHC3(1) sequence, the primer pair PHC3(For)983 and PHC3(Rev) was used (see Table S6). The PCR product was then cut with restriction enzymes *Bam*HI and *Xba*I and inserted into the pRS316-derived plasmid pCUP1-Sup35(NM)Om-CFP (unpublished), replacing the *Bam*HI and *Xba*I fragment, containing the sequence of the *Ogaetea methanolica* (*Om*) *SUP35NM* region.

The plasmid pGPD-PrP-YFP, expressing the mouse PrP protein from the strong constitutive *P_GPD_* promoter, was described earlier [54].

#### 4.4.2. Plasmids for the Analysis of PHC3 Aggregation in the C-DAG Assay 

Plasmids pVS72 and pVS105 encoding, respectively, the *S. cerevisiae* SUP35NM and SUP35M proteins, fused to the sequence coding for the CsgAss peptide (CsgA signal sequence), facilitating the export of the protein to the surface of bacterial cells, were kindly provided by A. Hochschild [24]. Plasmids C-DAG-PHC3(5) and C-DAG-PHC3(6) were constructed by fusing the sequences of the PHC3(5-1) and PHC3(6) isoforms, amplified, respectively, from the pCUP1-Sup35N-PHC3(5-1) and pCUP1-Sup35N-PHC3(6) plasmids by using primers PHC3(5)-NotI-For and PHC3(5)-XbaI-Rev, or PHC3(6)-NotI-For and PHC3(6)-XbaI-Rev (Table S2), and cut with *Not*I and *Xba*I, into the plasmid pVS105, hydrolyzed by the same enzymes. As a result, the respective PHC3 isoform-coding sequence replaced the original *SUP35NM* sequence in the resulting plasmids.

#### 4.4.3. Plasmids for the Analysis of PHC3 Aggregation in Human Cells

The plasmids pLenti-CMV-PHC3(5)-EGFP and pLenti-CMV-PHC3(6)-EGFP, containing sequences that encode the chimeric proteins PHC3(5-1)-EGFP and PHC3(6)-EGFP, both under the control of the cytomegalovirus (CMV) promoter, were constructed by PCR-amplifying the sequences coding PHC3 protein isoforms, respectively, from pCUPI-SUP35N-PHC3(5-1) and pCUPI-SUP35N-PHC3(6) with primers PHC3(5)-XbaI-For and PHC3(5)-BamHI+2-Rev, or PHC3(6)-XbaI-For and PHC3(6)-BamHI+2-Rev, including *Bam*HI and *Xba*I sites (Table S6), and first inserting them into the pJet1.2 vector (Thermo Fisher Scientific, Waltham, MA, USA) followed by digestion with *Bam*HI and *Xba*I restriction endonucleases, and the insertion of PHC3(5-1) and PHC3(6) fragments into the pLenti-CMV-GFP Hygro vector (656-4) [55], digested at the same sites.

### 4.5. Yeast Assays

#### 4.5.1. Phenotypic Detection of Sup35 Nucleation

The phenotypic assay for prion nucleation in yeast was conducted as described previously [41]. In brief, yeast strain GT409, which is [*psi*^−^][*pin*^−^] (that is, lacking any known prions), was transformed individually with each plasmid containing Sup35N (the prion domain of Sup35) fused with various PHC3 isoform-coding sequences, as well as with positive (pCUP1-Sup35N-Aβ_42_) and negative (pCUP1-Sup35N-Aβ_42_***) controls. Transformants were selected and patched on the medium lacking uracil (−Ura) and velveteen replica-plated onto the same medium supplemented with 150 µM CuSO_4_ to induce high-level expression from the *P_CUP1_* promoter. After two days of incubation, yeast cells were velveteen-replica-plated onto the medium lacking adenine (−Ade). The induction of the Sup35 prion ([*PSI*^+^]) was detected by growth on −Ade, typically after 7 days of incubation.

#### 4.5.2. Analysis of Aggregation by Fluorescence Microscopy

For this purpose, chimeric proteins obtained by the fusion of PHC3 isoforms to fluorescent proteins YFP or CFP were employed. Experiments were performed in the strain BY4742, which is prototrophic by adenine and, therefore, does not accumulate red pigment, sometimes interfering with fluorescence detection. Aβ_42_-YFP and Aβ_42_***-YFP were used as positive (aggregating) and negative (non-aggregating) controls, respectively. The expression of all constructs was induced by the addition of 150 µM of CuSO_4_. Pre-cultures were incubated overnight in liquid selective media with CuSO_4_, followed by inoculation into a fresh selective media with CuSO_4_, and grown until the OD_600_ was between 0.6 and 0.8. Fluorescence analysis was conducted using a confocal laser-scanning microscope (Leica TCS SP5, Leica Microsystems GmbH, Wetzlar, Germany). CFP-tagged proteins were detected using an excitation wavelength of 458 nm and an emission range of 461–510 nm. YFP and YFP-tagged proteins were detected using an excitation wavelength of 514 nm and an emission range of 518–580 nm.

### 4.6. C-DAG Assay

The C-DAG system assay was performed as described [24]. In brief, *E. coli* strain VS39 was transformed with respective plasmids and plated on an LB medium with ampicillin and chloramphenicol. The resulting transformants were patched on LB media containing Congo Red dye at a final concentration of 4 µg/mL, Amp (100 µ/mL), Cm (25 µg/mL), L-arabinose 0.2% (m/V), and 1 mM IPTG. Following a period of growth of 7–10 days at 20 °C, the bacterial cells were subjected to analysis using polarizing (Leica DMI 6000) and electron microscopy. For electron microscopy, bacterial cells were resuspended in 10 µL of distilled water and applied to copper grids coated with formvar (Agar Scientific, Rotherham, UK). Two minutes after the bacterial cells were bound to the grid surface, the sample was washed twice with distilled water, followed by contrasting the samples for two minutes in a 1% uranyl acetate solution. Electron microscopy was performed using the Jeol JEM-2100 microscope (JEOL Ltd., Tokyo, Japan).

### 4.7. Transfection and Viral Transduction of Human Cells

HEK293T cells were transfected with the pLenti-PHC3(5-1)-EGFP and pLenti-PHC3(6)-EGFP plasmids using polyethylenimine, following a procedure described earlier [56]. Human fibroblasts (hFBs) were transduced with lentiviruses encoding the CMV-PHC3(5-1)-EGFP and CMV-PHC3(6)-EGFP sequences. Lentiviruses were derived from the plasmids pLenti-PHC3(5-1)-EGFP and pLenti-PHC3(6)-EGFP. Lentiviral assembly and transduction were performed in S2 conditions, adhering to all the necessary safety precautions, as described previously [56].

### 4.8. Analysis of Protein Aggregation by Semi-Denaturing Agarose Gel Electrophoresis (SDD-AGE)

For protein analysis, yeast cultures containing plasmids with fluorescent tags were grown as described above in Section 4.5.2. Yeast cell lysates were prepared by disrupting yeast cells with glass beads, as described previously [57]. For this purpose, harvested cells were precipitated for 5 min at 2500 g, 4 °C, washed with ice-cold lysis buffer (150 mM NaCl, 50 mM Tris–HCl pH 7.5, 2 mM EDTA pH 8.0, 4 mM PMSF), precipitated again (5 min, 2500 g, 4 °C), and resuspended in 1 mL of lysis buffer with 1% Protease Inhibitor Cocktail (P8215, Sigma-Aldrich, St. Louis, MO, USA), followed by transfer to 1.5 mL microcentrifuge tubes, reprecipitation (5 min, 2500 g, 4 °C), and the removal of the supernatant. The pellet was resuspended in an equal volume of ice-cold lysing buffer containing protease inhibitors and mixed with an equal volume of 0.5 mm glass beads (G8772, Sigma-Aldrich, St. Louis, MO, USA), followed by intense mixing using a mixer (IKA, Wilmington, NC, USA), for 9–10 cycles of 20 s each, with intervals between cycles from 40 to 60 sec on ice. The upper fraction containing cell lysate was then transferred to new microtubes and centrifuged (5 min, 2500 g, 4 °C) to precipitate undegraded yeast cells and cell wall debris. The supernatant was then transferred to new tubes and used for gel electrophoresis as described below, or stored at −80 °C if necessary.

In the case of human HEK293T cells overexpressing EGFP, PHC3(5-1)-EGFP, or PHC3(6)-EGFP, protein lysates were prepared using the commercial M-PER™ Mammalian Protein Extraction Reagent (Thermo Fisher Scientific, Waltham, MA, USA) in accordance with the manufacturer’s instructions.

Semi-denaturing agarose gel electrophoresis (SDD-AGE) for the detection of protein aggregates was performed as described previously [58] with some modifications. An equal volume of 2× sample buffer (100 mM Tris–HCl pH 6.8, 20% glycerol, and 6% sodium lauryl sarcosinate, SLS) was added to each sample. Unboiled samples were then incubated in the sample buffer at room temperature (RT) for 10 min before being applied to the agarose gel, while preboiled samples were incubated in the boiling water bath for 10 min. Electrophoresis was performed using a 1.5% agarose gel containing 0.3% SLS, with an electric field strength of approximately 3 V/cm in TAE buffer (composed of 40 mM Tris–HCl, 20 mM acetic acid, 1 mM EDTA, and 0.3% SLS). After electrophoresis, proteins were transferred to a 0.45 μm PVDF membrane (Thermo Fisher Scientific, Waltham, MA, USA) via capillary blotting [59,60] and detected using antibodies specific to the GFP protein (also recognizing YFP), which were diluted to a ratio of 1:7000 (ab011, Evrogen, Moscow, Russia).

### 4.9. Immunocytochemistry (ICC)

To detect endogenous full-length PHC3 isoforms in human cells, an ab80612 antibody (Abcam, Cambridge, UK) was utilized, along with secondary antibodies conjugated with an Alexa647 fluorescent tag (ab150079) (Abcam, Cambridge, UK). HEK293T cells were seeded at a density of approximately 7000 cells per cm^2^ on round coverslips (13 mm diameter). The following day, the cells were fixed with 4% paraformaldehyde (Sigma-Aldrich, St. Louis, MO, USA) and permeabilized using 0.1% Triton X-100 for 15 min at room temperature. To enhance antibody binding to the antigens, the cells were washed thrice with PBS and heated to 80°C in antigen retrieval buffer (ab93678, Abcam, Cambridge, UK) for 20 min, followed by washing and incubation in a blocking solution containing 0.1% Triton X-100 and 4% donkey serum. Following three washes with PBS to remove the blocking solution, primary antibodies against the full-length PHC3 isoform (ab80612, dilution 1:300) were applied for 12 h at 4 °C. After several washes with PBS, secondary antibodies conjugated with Alexa Fluor 647 (ab150079, Abcam, Cambridge, UK, dilution 1:500) were added and incubated for 60 min at room temperature in the dark. The nuclei were then counterstained with Hoechst 33342 Ready Flow (Thermo Fisher Scientific, Waltham, MA, USA). Finally, after three additional washes with PBS, the cells were observed using confocal laser-scanning microscope Leica TSC SP5 (Leica Microsystems GmbH, Wetzlar, Germany).

### 4.10. Analysis of Differential Gene Expression

For the comparative analysis of differential gene expression, RNA from HEK293T cells overexpressing either EGFP or PHC3(5-1)-EGFP was extracted using the PureLink RNA Mini Kit (Invitrogen, Carlsbad, CA, USA), following the manufacturer’s protocol. The RNA quality and quantity were monitored using agarose gel electrophoresis, a NanoDrop, and a Bioanalyzer. In total, 12 sample libraries were prepared, including 6 biological replicates for each PHC3(5-1)-EGFP overexpressor and 6 biological replicates for the control EGFP overexpressor. The RNA-seq libraries were prepared from 1 µg of total RNA using the Illumina TruSeq stranded mRNA sample preparation kit. All sequencing was performed on Illumina HiSeq 2500 with a maximum read length of 2 × 101 bp. The paired-end library was prepared using the TruSeq stranded mRNA Sample Preparation kit following the manufacturer’s protocol.

Quality control of the raw sequence data was performed using the FastQC tool [61]. Reads were aligned to the human GRCh38.p13 reference genome using the STAR aligner [62]. Gene expression levels were quantified using the FeatureCounts tool as part of the Rsubread package (ver. 2.16.1) [63]. FeatureCounts assigns reads to genomic features, such as exons and genes, to produce count data representing the abundance of each gene in the samples. Three distinct DE analyses were performed to compare the different conditions using the DESeq2 package (ver. 1.42.1) [64]. All analyses and visualizations were conducted in RStudio (ver. 2023.09.1). ENSEMBL gene identifiers were mapped to gene symbols using the org.Hs.eg.db annotation package (ver. 3.18.0; available online: https://bioconductor.org/packages/release/data/annotation/html/org.Hs.eg.db.html; accessed on 26 December, 2024). Gene ontology (GO) enrichment analysis was conducted using the Clusterprofiler (ver. 4.10.1) [65] and Enrichplot (ver. 1.22.0) (available on: https://github.com/YuLab-SMU/enrichplot; accessed on 26 December, 2024) packages. Gene ontology (GO) enrichment analysis was performed on significantly differentially expressed genes. The gene ratio (Figure 5G) was determined as the number of differentially expressed genes associated with a specific GO term, divided by the total number of differentially expressed genes with a *p*-adjusted value, *p_adj_* < 0.01. For determining the *p_adj_* value, we used the Benjamini–Hochberg (BH) method considering a false discovery rate, FDR [65]. Differential gene expression and GO analysis and visualization were performed in RStudio software (ver. 2023.09.1+494).

## 5. Conclusions

Short isoforms of PHC3 possess amyloidogenic and prionogenic properties, leading to the cytosolic aggregation and potential trapping of full-length protein. This may produce an aggregation-based regulatory mechanism, modulating chromatin packaging and gene transcription.

## Data Availability

The original contributions presented in this study are included in the article/Supplementary Materials. Further inquiries can be directed to the corresponding author(s).

## Supplementary Materials

The supporting information can be downloaded at: https://www.mdpi.com/article/10.3390/ijms26041512/s1.
