# Supplementary material for: Prion-like Properties of Short Isoforms of Human Chromatin Modifier PHC3
Source: Int J Mol Sci. 2025 Feb 11;26(4):1512. doi: 10.3390/ijms26041512 (PMC11855497; doi:10.3390/ijms26041512)
Supplement: Supplementary file 1 [file ijms-26-01512-s001.zip › 2025 PHC3 Dataset Descriptions.pdf]

Dataset descriptions

Dataset S1. Gene counts for expression analysis. This dataset demonstrates the raw number of reads for each gene across all samples after aligning on the human GRCh38.p13 reference genome using the STAR aligner. Gene_id - the unique gene identifier from the ENSEMBL database; S1-S6_EGFP – samples with ectopic EGFP overexpression; S7-S12_PHC3(5-1)-EGFP – samples with ectopic PHC3(5-1)-EGFP overexpression.

Dataset S2. List of differentially expressed genes. This dataset shows the results of differential gene expression analysis between HEK293T cells with ectopic overexpression of PHC3(5-1)-EGFP compared to the same cell line with ectopic EGFP overexpression. baseMean - The average normalized count of a gene across all samples; log2FoldChange - The log base 2 of the fold change in expression between two conditions; lfcSE (Log2 Fold Change Standard Error) - The standard error of the log2 fold change estimate; stat - The Wald test statistic for the log2 fold change; pvalue - The p-value for the test of differential expression; padj (adjusted p-value) - The p-value after correction for multiple testing (Benjamini-Hochberg correction); ENSEMBL - The unique gene identifier from the ENSEMBL database; SYMBOL - Readable gene symbol or name

Dataset S3. Expression of the ubiquitin-proteasome system genes. This dataset presents the differentially expressed genes associated with the proteasome-mediated ubiquitin-dependent protein catabolic process; GO:0043161 gene ontology term identified during differential gene expression analysis. ENSEMBL - the unique gene identifier from the ENSEMBL database; SYMBOL - readable gene symbol or name; baseMean - the average normalized count of a gene across all samples; log2FoldChange - the log base 2 of the fold change in expression between two conditions; lfcSE (Log2 Fold Change Standard Error) - the standard error of the log2 fold change estimate; stat - The Wald test statistic for the log2 fold change; pvalue - the p-value for the test of differential expression; padj (adjusted p-value) - the p-value after correction for multiple testing (Benjamini-Hochberg correction).
