# Supplementary material for: Prion-like Properties of Short Isoforms of Human Chromatin Modifier PHC3
Source: Int J Mol Sci. 2025 Feb 11;26(4):1512. doi: 10.3390/ijms26041512 (PMC11855497; doi:10.3390/ijms26041512)
Supplement: Supplementary file 1 [file ijms-26-01512-s001.zip › ijms-3424420-supplementary.pdf]

Prion-like Properties of Short Isoforms of Human Chromatin Modifier PHC3

Daniil Kachkin, Andrew A. Zelinsky, Nina V. Romanova, Konstantin Y. Kulichikhin, Pavel A. Zykin, Julia I. Khorolskaya, Zachery J. Deckner, Andrey V. Kajava, Aleksandr A. Rubel, Yury O. Chernoff

Supplementary Information

Table S1. Amyloidogenic and aligned regions of PHC proteins

Protein	Motifs predicted by ArchCandy	Motifs predicted by AmylPred2	Aligned regions
PHC1	49-80; 180-229; 242-275; 331-353; 386-438; 528-545	55-58; 130-134; 173-178; 397-403; 618-628; 733-743; 946-951;972-975; 996-999	16-76; 102-154; 720-758 (HD1); 940-1004 (SAM)
PHC2	70-131; 149-187; 523-548	60-62; 190-196; 451-458; 496-497; 524-528; 568-578; 800-803	32-112; 137-195; 555-593 (HD1); 794-858 (SAM)
PHC3*	48-61; 73-112	59-64; 189-195; 200-204; 254-259; 423-427; 549-553; 599-600; 713-723; 755-759; 931-933; 963-964	40-114; 138-194; 701-738 (HD1); 931-995 (SAM)

Numbers indicate amino acid positions for each protein.

* Numbering corresponds to the maximal length isoform PHC3(7).

Table S2. Detection of aggregates in yeast cells by fluorescence microscopy (Data for Fig. 3C).

Construct	Cells with aggregates			Total number of cells with fluorescence
	Number	%	SE*, %	
Aβ42-YFP	273	98.9	± 0.6	276
$A\beta42^{***}$-YFP	0	0	+ 0.2	523
PHC3(1)-CFP	201	94.8	± 1.5	212
PHC3(5-1)-YFP	324	59.0	± 2.1	549
PHC3(6)-YFP	147	53.1	± 3.0	277

Data are combined from 4 independent cultures in each case.
*SE – standardized error of proportion. (Same designation is in Table S3.)

Table S3. Colocalization of aggregates formed by short and full-length isoforms of PHC3 in yeast cells. (Data for Fig. 3D.)

Constructs	Cells with aggregates			Total number of cells with both types of aggregates
	Number	%	SE*, %	
YFP-PHC3(5-1) + PHC3(1)-CFP	99	75.6	± 3.8	131
YFP-PHC3(6) + PHC3(1)-CFP	69	57.5	± 4.5	120

Table S4. Localization of full-length PHC3 in cells with overexpression of truncated PHC3 isoforms. (Data for Fig. 5D.)

Constructs	Cells with cytosolic full-length PHC3			Total number of cells analyzed
	Number	%	SE*, %	
No vector	13	7.0	± 1.9	187
CMV-EGFP	8	7.6	± 2.6	106
CMV-PHC3(5-1)-EGFP	7	10.5	± 3.7	67
CMV-PHC3(6)-EGFP	10	12.1	± 3.6	83

Table S5. Plasmid and viral vectors used in this study.

Vector	Promoter/Gene	Marker(s)	Source
pCUP1-YFP(-2)-INTctd	P_{CUP1}-YFP-INTctd	*2μ LEU2* Amp^R	This study
pCUP1-Sup35(NM)Om-CFP	P_{CUP1}-*SUP35(NM)*$_{Om}$*-CFP*	*2μ URA3* Amp^R	This study
pCUP1-Sup35N-PHC3(5-1)	P_{CUP1}-*SUP35N-PHC3(5-1)*	*CEN URA3* Amp^R	This study
pCUP1-Sup35N-PHC3(5-2)	P_{CUP1}-*SUP35N-PHC3(5-2)*	*CEN URA3* Amp^R	This study
pCUP1-Sup35N-PHC3(6)	P_{CUP1}-*SUP35N-PHC3(6)*	*CEN URA3* Amp^R	This study
pCUP1-Sup35N-Aβ42	P_{CUP1}-*SUP35N-Aβ42*	*CEN URA3* Amp^R	[41]
pCUP1-Sup35N- $A\beta42^{***}$	P_{CUP1}-*SUP35N-*$A\beta42^{***}$	*CEN URA3* Amp^R	[41]
pCUP1-YFP-PHC3(5-1)	P_{CUP1}-*YFP-PHC3(5-1)*	*2μ LEU2* Amp^R	This study
pCUP1-YFP-PHC3(6)	P_{CUP1}-*YFP-PHC3(6)*	*2μ LEU2* Amp^R	This study
pCUP1-PHC3(1)-CFP	P_{CUP1}-*PHC3(1)-CFP*	*2μ URA3* Amp^R	This study
pCUP1-Aβ42-YFP	P_{CUP1}-*Aβ42-YFP*	*2μ LEU2* Amp^R	This study
pCUP1-$A\beta42^{***}$-YFP	P_{CUP1}-$A\beta42^{***}$*-YFP*	*2μ LEU2* Amp^R	This study
pGPD-PrP-YFP	P_{GPD}-*Prnp-YFP*	*2μ URA3* Amp^R	[54]
pC-DAG-CsgAss-PHC3(5-1)	P_{BAD}-*CsgAss-PHC3(5-1)*	Amp^R	This study
pC-DAG-CsgAss-PHC3(6)	P_{BAD}-*CsgAss-PHC3(6)*	Amp^R	This study
pVS72	P_{BAD}-*CsgAss-Sup35NM*	Amp^R	[24]
pVS105	P_{BAD}-*CsgAss-Sup35M*	Amp^R	[24]
pLenti-CMV-GFP Hygro (656-4)	P_{CMV}-*EGFP*	Hyg^R Amp^R	[55]
LV-CMV-EGFP Hygro (656–4)	P_{CMV}-*EGFP*	Hyg^R	This study
pMD2.G	P_{CMV}-*VSV-G*	Amp^R	Didier Trono (Addgene #12259)
psPAX2	P_{CAG}-*GAG, Pol, Rev, Tat*	Amp^R	Didier Trono (Addgene #12260)
pLenti-CMV-PHC3(5-1)-EGFP	P_{CMV}-*PHC3(5-1)-EGFP*	Hyg^R Amp^R	This study
pLenti-CMV-PHC3(6)-EGFP	P_{CMV}-*PHC3(6)-EGFP*	Hyg^R Amp^R	This study

Table S6. Primers used in this study.

Primer	Sequence 5′-3′
PHC3(5)-SfiI-For	TAGGCCATTATGGCCGCGGAAGCGGAATTTAAG
PHC3(5,6)-SfiI-Rev	GGCCGAGGCGGCCTTAAAATTTTAAATCCAG
PHC3(6)-SfiI-For	TAGGCCATTATGGCCGATACTGAACCAAACCC
PHC3(5)-NotI+1-For	ATGCGGCCGCAATGGCGGAAGCG
PHC3(5,6)-XbaI-Rev	GGACTCTAGATTAAAATTTTAAATCCAGAAAAAAAAAATTTAGGG
PHC3(6)-NotI-For	ATGCGGCCGCAATGGATACTGAACC
PHC3(5)-XbaI-For	CATCTAGAATGGCGGAAGCGGAATTTAAGG
PHC3(5,6)-BamHI+2-Rev	GTGGATCCGCAAATTTTAAATCCAGAAAAAAAAAATTTAG
PHC3(6)-XbaI-For	ACTCTAGAATGGATACTGAACCAAACCCG
PHC3(For)983	AAGGATCCATGGATACTGAACCAAACCCGGGAACATCTTCTGTG
PHC3(Rev)	AATCTAGAAGATTCCTTCAGAGAGTTGATGCGTGCACAGATCTT
